# *SODB1* is essential for *Leishmania major* infection of macrophages and pathogenesis in mice

**DOI:** 10.1371/journal.pntd.0006921

**Published:** 2018-10-29

**Authors:** Bennett J. Davenport, Casey G. Martin, Stephen M. Beverley, David J. Orlicky, Andres Vazquez-Torres, Thomas E. Morrison

**Affiliations:** 1 Department of Immunology and Microbiology, University of Colorado School of Medicine, Aurora, Colorado, United States of America; 2 Department of Pathology, University of Colorado School of Medicine, Aurora, Colorado, United States of America; 3 Department of Molecular Microbiology, Washington University School of Medicine, Saint Louis, Missouri, United States of America; McGill university, CANADA

## Abstract

*Leishmania* species are sand fly-transmitted protozoan parasites that cause leishmaniasis, neglected tropical diseases that affect millions of people. *Leishmania* amastigotes must overcome a variety of host defenses, including reactive oxygen species (ROS) produced by the NADPH oxidase. *Leishmania* species encode three superoxide dismutases (SODs): the mitochondrial SODA and two glycosomal SODs (SODB1 and SODB2). SODs are metalloenzymes that function in antioxidant defense by converting superoxide to oxygen and hydrogen peroxide. Here, we investigated a role for SODB1 in *Leishmania* infection of macrophages and virulence in mice. We found that a single allele deletion of *SODB1* (*SODB1*/Δ*sodb1*) had minimal effects on the replication of axenically-grown *L*. *major* promastigotes or differentiation to infective metacyclic promastigotes. Disruption of a single *SODB1* allele also did not affect *L*. *donovani* differentiation to amastigotes induced axenically, or the replication of axenically-grown *L*. *donovani* promastigotes and amastigotes. In contrast, the persistence of *SODB1*/Δ*sodb1 L*. *major* in WT macrophages was impaired, and the development of cutaneous lesions in *SODB1*/Δ*sodb1 L*. *major*-infected C57BL/6 and BALB/c mice was strongly reduced. The reduced disease severity in mice was associated with reduced burdens of *SODB1*/Δ*sodb1 L*. *major* parasites in the foot at late, but not early times post-inoculation, as well as an impaired capacity to disseminate from the site of inoculation. Collectively, these data suggest that *SODB1* is critical for *L*. *major* persistence in macrophages and virulence in mice.

## Author summary

*Leishmania* protozoan parasites are the causative agents of leishmaniasis, neglected tropical diseases with clinical manifestations ranging from self-limiting cutaneous lesions to invasive disease involving multiple organs. During blood-feeding by an infected sand fly, *Leishmania* parasites are transmitted to human hosts where they establish long-term infection in cells such as macrophages. An improved understanding of the mechanisms by which *Leishmania* parasites replicate and persist in macrophages may identify new therapeutic targets for the treatment of leishmaniasis. Using genetically-altered *Leishmania* parasites, cell-culture approaches, and a mouse model of *Leishmania* infection and disease, we found that a normal level of *Leishmania* superoxide dismutase B1 (SODB1) is necessary for *Leishmania major* persistence in macrophages and for virulence in mice. This work contributes new knowledge about the pathogenic mechanisms of *Leishmania major* infection and suggests that SODB1 may be an efficacious therapeutic target.

## Introduction

*Leishmania* parasites are sand fly-transmitted protozoan parasites that cause leishmaniasis in humans and other animals. Depending on the infecting *Leishmania* species, a spectrum of clinical manifestations can occur, ranging from self-limiting cutaneous lesions to invasive disease involving multiple organs. Visceral leishmaniasis (VL), the most severe form of the disease, is a progressive and chronic illness that, if left untreated, is nearly always fatal [1].

More than 350 million people are at risk of contracting leishmaniasis [2], and the disease disproportionately affects the poor. It is estimated that 20,000 to 40,000 deaths occur from leishmaniasis each year, with the Global Burden of Disease Study 2015 estimating more than 20,000 deaths from VL [3,4]. There are no approved vaccines against *Leishmania* infection. Although existing treatments can be effective, drug toxicity, poor access to health care services, and the development of resistance are significant complications in the treatment of leishmaniasis.

In sand flies, *Leishmania* parasites replicate extracellularly in the digestive tract as flagellated promastigotes [5]. During blood-feeding by an infected sand fly, promastigotes are deposited into the skin where they are taken up by phagocytic cells such as neutrophils and macrophages [6]. In macrophages, *Leishmania* parasites differentiate into amastigotes, a morphologically and metabolically distinct infective form, and replicate within parasitophorous vacuoles [7–9]. A better understanding of the mechanisms by which *Leishmania* parasites replicate and persist in macrophages may identify new therapeutic targets for the treatment of leishmaniasis.

Superoxide dismutases (SODs) are metalloenzymes that function in antioxidant defense by converting superoxide to oxygen and hydrogen peroxide [10]. SODs are a large family of proteins that use the metals manganese (Mn), iron (Fe), nickel (Ni), or copper and zinc (Cu/Zn) as cofactors [11]. FeSODs, which are structurally similar to MnSODs typically found in mitochondria, are dimers with each active site containing a single iron bonded to three histidines, one aspartate, and one water molecule [11]. Multiple FeSOD genes are encoded by *Leishmania* parasites, including *SODA*, *SODB1*, and *SODB2*. The *SODB1* and *SODB2* genes are arranged in tandem on the same chromosome, and sequence comparisons revealed greater than 92% identity across multiple *Leishmania* species (*L*. *chagasi*, *L*. *donovani*, and *L*. *major*) [12]. In addition, the amino acid sequences of SODB1 and SODB2 are ~90% identical, with the major difference occurring at the 3′ end that is extended by ~13 amino acids in SODB2 [12].

In *L*. *chagasi*, SODB1 and SODB2 proteins are targeted by three amino acids at the C-terminus to the glycosome [12], a unique membrane-bound organelle found in kinetoplastid parasites that functions in glycolysis, β-oxidation of fatty acids, and other metabolic activities [13,14]. A single allele knockout of *SODB1* resulted in decreased survival of *L*. *chagasi* in differentiated U937 cells and decreased survival of parasites exposed to paraquat, which increases intracellular superoxide levels [12]. In addition, expression of an antisense transcript against FeSOD in *L*. *tropica* and *L*. *donovani*, which may have effected expression of SODA, SODB1, and/or SODB2, resulted in enhanced sensitivity of parasites to the superoxide generator menadione and H_2_O_2_ in axenic cultures as well as reduced parasite survival in murine macrophages [15]. These findings suggest that FeSODs are important for the replication and/or survival of *Leishmania* parasites in macrophages and for protection against oxidative stress.

In this study, we investigated the role of *SODB1* in promoting *L*. *major* infection in murine macrophages and pathogenesis in a mouse model of cutaneous leishmaniasis. Using conventional gene disruption techniques, we generated *L*. *major* and *L*. *donovani* parasites harboring a single allele deletion of the *SODB1* gene (*SODB1*/Δ*sodb1)*. We show that a single allele deletion of *SODB1* does not affect promastigote or amastigote growth in cell culture, promastigote metacyclogenesis, or amastigote differentiation. *In vitro* infection assays performed in bone marrow-derived macrophages (BMDMs) from WT mice revealed that *SODB1*/Δ*sodb1 L*. *major* parasites were unable to persist at levels of WT *L*. *major*. Furthermore, complementation of *SODB1*/Δ*sodb1 L*. *major* with exogenous expression of the SODB1 protein rescued attenuation in BMDMs. Experiments in a mouse model of *Leishmania* pathogenesis showed that WT mice infected with *SODB1*/Δ*sodb1 L*. *major* developed less severe lesions compared with mice infected with WT *L*. *major*. The attenuated virulence of *SODB1*/Δ*sodb1 L*. *major* in mice was associated with reduced parasite burdens in the inoculated foot and impaired dissemination of parasites to the draining lymph node. Collectively, these findings suggest that SODB1 is essential for *L*. *major* infection and virulence and further support SODB1 as a potential therapeutic target for the treatment of *Leishmania* infection and disease.

## Methods

### Ethics statement

This study was conducted in accordance with the recommendations in the Guide for the Care and Use of Laboratory Animals and the American Veterinary Medical Association (AVMA) Guidelines for the Euthanasia of Animals. All animal experiments conducted at the University of Colorado Anschutz Medical Campus were performed with the approval of the Institutional Animal Care and Use Committee (IACUC) at the University of Colorado School of Medicine (Assurance Number: A3269-01) under protocol 00222.

### Chemicals and reagents

Adenine (A2786), hemin (H9039), d-biotin (B4639), folic acid (F8758), 100x RPMI vitamin mix (R7256), 100x RPMI amino acid mix (R7131), hygromycin B (H3274), phleomycin (P9564), Wright-Giemsa Stain (WG16) and proteinase K (P4250) were acquired from Sigma-Aldrich. M199 with Hanks’ salts (M2852) was acquired from US Biological Life Sciences. L-biopterin (11.203) was acquired from Schircks Laboratories (Switzerland). G418 (04 727 878 001) was acquired from Roche. Peanut Agglutinin (L107025) was acquired from Vector Laboratories. AquaVi-421 LIVE/DEAD fixable cell dye (L34955) and SuperScript IV First Strand Synthesis System (18091050) were acquired from Life Technologies. Ambion PureLink RNA mini kit (12183025), random primers (48190011), TRIzol Reagent (15596018), and PureLink DNase (12185010) were acquired from Life Technologies.

### *Leishmania* parasites and parasite culture

*L*. *major* strain NIH Friedlin V (MHOM/IL/80/FN) was obtained from BEI Resources (NR-48815). *L*. *donovani* 1S2D sub-strain LdBob was generated as previously described [16]. *L*. *major* promastigotes were maintained at 26°C in complete M199 culture media (1x M199, 40 mM HEPES pH 7.4, 100 μM adenine, 4 μM biotin, 7.6 μM hemin, 50 μg/ml penicillin, 50 U/ml strep, 8 μM L-biopterin and 10% FBS). LdBob promastigotes were maintained at 26°C in complete proM199:RPMI culture media (1x M199, 25 mM HEPES pH 6.9, 12 mM NaHCO_3_, 1x L-glutamine, 0.1 mM adenine, 23 μM folic acid, 7.6 μM hemin, 50 U/ml penicillin, 50 μg/ml streptomycin, 1x RPMI vitamin mix and 10% FBS). To induce axenic amastigote differentiation, LdBob promastigotes were seeded into amaM199:RPMI culture media (1x M199, 27.5 mM MES, 25 mM NaHCO3, 1x L-glutamine, 0.1 mM adenine, 23 μM folic acid, 7.6 μM hemin, 50 U/ml penicillin, 50 μg/ml streptomycin, 1x RPMI vitamin mix, and 13% FBS), adjusted to pH 5.5 with HCl and maintained at 37°C with 5% CO_2_ as previously described [16]. To enumerate parasite cell numbers in culture, cells were fixed in 2% paraformaldehyde (PFA) and counted via hemocytometer.

### Generation of *L*. *major* and *L*. *donovani SODB1* single allele deletion strains

To generate *SODB1/Δsodb1 L*. *major* and *L*. *donovani* parasites, gene deletion constructs were developed. *L*. *major* and *L*. *donovani* sequences were acquired from the Kyoto Encyclopedia of Genes and Genomes (KEGG) (http://www.genome.jp/kegg-bin/show_organism?org=lma and http://www.genome.jp/kegg-bin/show_organism?org=ldo), including coding regions and flanking intergenic regions (IGRs) for *L*. *major SODB1* (*XM_001685449*) and *SODB2* (XM_001685450), as well as *L*. *donovani SODB1* (*XM_003863559)* and *SODB2* (XM_003863560). The IGRs upstream and downstream of the *SODB1* coding sequence were amplified from *L*. *major* and *L*. *donovani* genomic DNA (gDNA) by PCR (**S1 Table**). The 5′ arm for *L*. *major* and *L*. *donovani* constructs contained the IGR immediately upstream (1,428 bp or 1,064 bp, respectively) of the *SODB1* start codon. The IGR for the 3′ arm of the *L*. *major* and *L*. *donovani* constructs contained the IGR immediately downstream of the *SODB1* stop codon (1,064 bp or 1,303 bp, respectively). The 5′ and 3′ arm amplicons were digested and sequentially cloned into the pBluescript SK(+) vector (pSK(+)-*sodb1*Lm or pSK(+)-*sodb1*Ld). G418 or phleomycin drug resistance genes were subcloned between the 5′ and 3′ arms of the pSK(+)-*sodb1*Lm and pSK(+)-*sodb1*Ld targeting constructs. Prior to transfection, pSK(+)-*sodb1*Lm and pSK(+)-*sodb1*Ld targeting constructs were linearized by PvuI digest and column purified, yielding a 1,050 bp fragment of pSK(+) plasmid backbone, and a 5,350 bp fragment containing the *SODB1* targeting construct DNA.

Transfection was performed as previously described [17]. Briefly, mid-log phase promastigotes were resuspended at 2 x 10^8^ cells/ml in cytomix electroporation buffer (120 mM KCl_2_, 150 μM CaCl_2_, 10 mM KH_2_PO_4_, 25 mM HEPES pH 7.6, 2 mM EDTA and 5 mM MgCl_2_). Ten μg of linearized pSK(+)-*sodb1*Lm or pSK(+)-*sodb1*Ld was added to 500 μl of cell suspension, parasite-DNA mixtures were electroporated in 4 mm cuvettes (two pulses of 1500V/25 μF), and transferred to 10 ml culture for 24 h in the absence of selection. Transfectants were selected on M199:Lmaj or proM199:RPMI agar culture plates containing respective drug for *L*. *major* or *L*. *donovani* transfectants, respectively. For drug selection, phleomycin (10 μg/ml), G418 (10 μg/ml), and/or hygromycin (50 μg/ml) were added to selection plates, and all subsequent cultures. Drug-resistant clones were genotyped for gene deletion by PCR reactions designed to amplify fragments that span the endogenous genomic locus and plasmid construct boundaries (**S1 Fig**). All PCR fragments were sequenced to confirm precise construct integration within the *SODB1* genomic locus. All parasite parent strains and clones were subsequently passaged through BALB/cJ mice to maintain virulence as previously described [18].

### *Leishmania* SODB1 and SODB2 gene expression analysis by quantitative RT-PCR

Log-phase promastigotes (*L*. *major* and *L*. *donovani*) or amastigotes (*L*. *donovani*) were homogenized in TRIzol reagent (Life Technologies) and total RNA was isolated using a PureLink RNA mini kit (Life Technologies). To limit DNA contamination of the RNA samples, an on-column DNase digestion step was performed. Full-length cDNA was generated from purified RNA using SuperScript IV reverse transcriptase and random primers. Quantitative PCR was performed on cDNA using *SODB1*- or *SODB2*-specific forward and reverse primers and TaqMan probes (**S1 Table**). For quantification, standard curves were generated from plasmids encoding *SODB1* or *SODB2* coding sequences. To ensure amplification specificity, no template and no reverse transcription controls for all samples were run in parallel.

### *L*. *major* and *L*. *donovani* morphological analysis

Log-phase parasite cultures were collected and fixed in 2% PFA. Cell suspensions were washed in H_2_O, pipetted onto glass coverslips, and air-dried. Dried coverslips were subjected to Wright-Giemsa stain for 2 min, washed with H_2_O for 5 min, and air-dried. Coverslips were mounted with Permount Mounting Media (Fisher SP15) and visualized on a Zeiss AxioPlan II microscope at 100x magnification.

#### Parasite viability assay and in vitro stress assays

Parasite viability was determined using AquaVi-421 and staining procedures according to the manufacturers guidelines (ThermoFisher Scientific). Briefly, 1 x 10^6^ parasites were stained in 100 uL of AquaVi-421 (stock solution diluted 1:2,000 in PBS) for 30 min at room temperature (RT). Following incubation, parasites were washed twice in PBS and fixed in 2% PFA for 15 min at RT. Following PFA fixation, cells were washed twice in PBS, and acquired on a BD Fortessa X-20 flow cytometer. Susceptibility of parasites to oxidative stress was assessed by exposure to H_2_O_2_ (H325; Fisher) or the superoxide producing compound menadione (M5625; Sigma). For short-term susceptibility assays, 5 x 10^5^ late-log phase parasites per well were seeded in 96-well plates in standard culture media and exposed to vehicle or graded doses of H_2_O_2_ (2 mM to 0.25 mM) for 4 h. Viability was quantified by flow cytometry using an AquaVi-421-based viability assay. For longer-term effects of oxidative stress induced by menadione, stationary phase promastigotes were seeded into 24-well plates in a volume of 1 mL at a concentration of 1 x 10^5^ parasites/mL, and allowed to culture for 24 h. Cultures were treated with vehicle or graded doses of menadione (2 μM to 0.5 μM), and viability was quantified at 6 days post-treatment as described above. Menadione was dissolved and diluted in DMSO, and volumes of menadione added to *Leishmania* cultures during all experiments were kept below 0.1%. Analysis of flow cytometry data was conducted using FlowJo (TreeStar) software.

### *SODB1/Δsodb1 L*. *major* complementation with N-terminally HA-tagged *SODB1*

The *Leishmania* gene overexpression construct pXG-*HYG* (pXG) was used for episomal gene overexpression analysis as previously described [19–21]. The *L*. *major SODB1* coding sequence was amplified by PCR using a 5′ primer containing the human influenza hemagglutinin (HA) sequence (**S1 Table**). Amplified (HA)-*SODB1* was then cloned into pXG. *SODB1/Δsodb1 L*. *major* parasites were transfected with pXG or pXG-(HA)-*SODB1* plasmids by electroporation and drug resistant clones were isolated. Overexpression of (HA)-*SODB1* was confirmed in whole cell lysates of stationary phase parasites by SDS-PAGE and Western blot analysis using primary anti-HA (clone HA-7) and secondary goat-αmouse IgG-HRP (Fisher 31430). Equal protein loading was confirmed by western blot analysis using anti-α-Tubulin (clone B-5-1-2).

### Macrophage infections

To generate BMDMs, bone marrow cells were aseptically flushed from mouse femurs and tibias, subjected to red blood cell lysis, and plated in DMEM (Gibco; 11995–065) supplemented with 10% FBS, 5% horse serum, and 20% L-cell conditioned media for 7 days. BMDMs were plated in 24-well plates at 5 x 10^5^ cells per well and cultured for 24 h. Cell cultures were inoculated with stationary phase *L*. *major* promastigotes or PNA^-^ metacyclic promastigotes at an MOI of 10 parasites/BMDM or 1 parasite/BMDM, respectively. Plates were centrifuged for 5 min at 200 x g to synchronize infection and incubated at 37°C for 4 h. After the 4 h infection, free parasites were removed by washing three times with PBS, and cultured in DMEM supplemented with 10% FBS and 100 U/mL penicillin/streptomycin.

### Genomic DNA isolation and parasite DNA quantification by qPCR

Isolation of gDNA from axenic parasite cultures or from infected BMDMs was carried out by proteinase K digestion and isopropanol precipitation. Briefly, cells were resuspended in 400 μl DNA lysis buffer (0.1 M Tris pH 8.0, 0.2 M NaCl, 5 mM EDTA, 0.4% SDS, and 0.2 mg/ml proteinase K) and incubated at 56°C overnight. Following digestion, gDNA was isolated by isopropanol precipitation and resuspended in H_2_O. To quantify total parasite numbers by qPCR, forward and reverse primers and a TaqMan probe directed against the *L*. *major* ubiquitin hydrolase (*UbHyd;* XM_003722265) gene were used (**S1 Table**). Total parasite genomes were extrapolated from a standard curve generated from *L*. *major* gDNA. To quantify total macrophage genomes, forward and reverse primers and a TaqMan probe directed against the C57BL/6J *gapdh* gene (BC023196) were used (**S1 Table**). Total genomes were extrapolated from a standard curve generated from gDNA isolated from BMDMs.

### Mouse infections

Eight week-old male and female WT C57BL/6J (000664) and eight week-old female WT BALB/cJ (000651) mice were purchased from Jackson Laboratories. Eight week-old *Nos2*^-/-^ C57BL/6J (002609) mice were obtained from Jackson Laboratories and bred in house, and combinations of male and female *Nos2*^*-/-*^ mice were included in all studies. Infectious metacyclic *L*. *major* promastigotes were isolated by agglutination assay as previously described [22,23]. Briefly, stationary phase promastigotes were washed twice in PBS, resuspended in 50 μg/ml peanut agglutinin (PNA) in PBS, and incubated at RT for 30 min. Agglutinated parasites were pelleted by centrifugation at 40 x g for 5 min, and infectious metacyclic promastigotes remaining in the supernatant were retrieved and washed twice in PBS. 10^4^ infectious metacyclic promastigotes in 40 μl of PBS were inoculated into the dorsal side of the right hind foot. Lesion development was assessed by measuring foot height and width using digital calipers. The area represents the product of the height and width, and the % of initial area represents the quotient (area of dpi/area of initial).

### Parasite burden analysis by limiting dilution assay

To quantify parasite burden in mouse tissues, limiting dilution assay of tissue homogenates was performed as previously described [24–26]. Briefly, the foot (including toes and skin) and the draining popliteal lymph node (popLN) were homogenized in M199:Lmaj media with a Roche MagNA Lyser. Tissue homogenates were seeded in duplicate in 96-well plates in a 4-fold dilution series and incubated at 26°C. At 7–14 days, the last dilution that contained motile promastigotes was determined.

### Tissue sections and histopathology

Inoculated foot tissue was harvested from mice infected with WT or *SODB1/Δsodb1 L*. *major* into 10% neutral buffered formalin. Tissue was processed, paraffin-embedded, and longitudinal tissue sections were stained with hematoxylin and eosin (H&E). H&E stained sections were scored for inflammation. The “extent of inflammation” was determined by the number of 100x fields required to cross the area of inflammation in a tilling fashion (each 100x field was assigned a value of 1). The “relative quantity” of inflammation was determined by examining the dermis and hypodermis tissues and estimating whether inflammatory cells filled 0%, 5%, 10%, 25%, or ≥50% (and assigned values of 0, 1, 2, 3, 4, 5) of the space between the normal connective tissue cells. The total inflammation was determined by multiplying the “extent of inflammation” by the “relative quantity” of inflammation. Histologic images were captured at the tissue site showing the highest degree of inflammation using an Olympus BX51 microscope equipped with a 4MP Macrofire digital camera (Optronics) and using the PictureFrame Application 2.3 (Optronics). All images were processed identically by Photoshop (Adobe Systems Inc., Mountain View, CA).

### Data handling and statistical analysis

All data handling, graphical representation, and statistical analysis were conducted using GraphPad Prism 6.0. Graphical representations of all data are expressed as the mean +/- the standard error of the mean (SEM), and statistical significance was evaluated using a two-tailed unpaired *t-*test, one-way ANOVA with Tukey’s multiple comparison test, or two-way ANOVA with Bonferroni’s multiple comparison test as indicated in the figure legends. Where applicable, variances in data were deemed significant with a *P* value < 0.05, and asterisks indicates the degree of significance (*P < 0.05, **P < 0.01, ***P < 0.001).

## Results

### *SODB1* gene disruption in *L*. *major* and *L*. *donovani*

To investigate the role of *SODB1* in *Leishmania* infection and pathogenesis, we generated *Leishmania* parasites with a disrupted *SODB1* allele. DNA constructs encoding a G418 resistance gene (*NEO*) flanked by IGR sequences upstream and downstream of the *SODB1* coding region were used to replace the complete endogenous *SODB1* coding region via homologous recombination (**S1A Fig**). Selection of *L*. *major* parasites carrying single allele disruption of the *SODB1* gene was readily achieved (**S1B Fig**). Precise integration of the targeting construct was confirmed using PCR primer combinations that specifically amplify products from either the *SODB1* WT genomic locus, or products that are unique to specific construct integration within the *SODB1* genomic locus on chromosome 32 (**S1B and S1C Fig**). Consistent with previous studies with *L*. *chagasi* parasites [12], repeated attempts to delete the second *SODB1* allele by homologous recombination were unsuccessful. To confirm these findings, we successfully replaced a single *SODB1* allele with a G418 drug resistance cassette in the *L*. *donovani* 1S2D sub-strain LdBob using similar gene disruption techniques as in *L*. *major* (**S1C Fig**). Similar to our observations using *L*. *major*, repeated attempts to delete the second *SODB1* allele in *L*. *donovani* parasites by homologous recombination also were unsuccessful. These data, together with previous reports, suggest that SODB1 is essential for the viability of promastigotes of multiple species of *Leishmania* parasites.

### *SODB1/Δsodb1 L*. *major* and *L*. *donovani* promastigotes grow and differentiate into amastigotes

**Fig 1:**
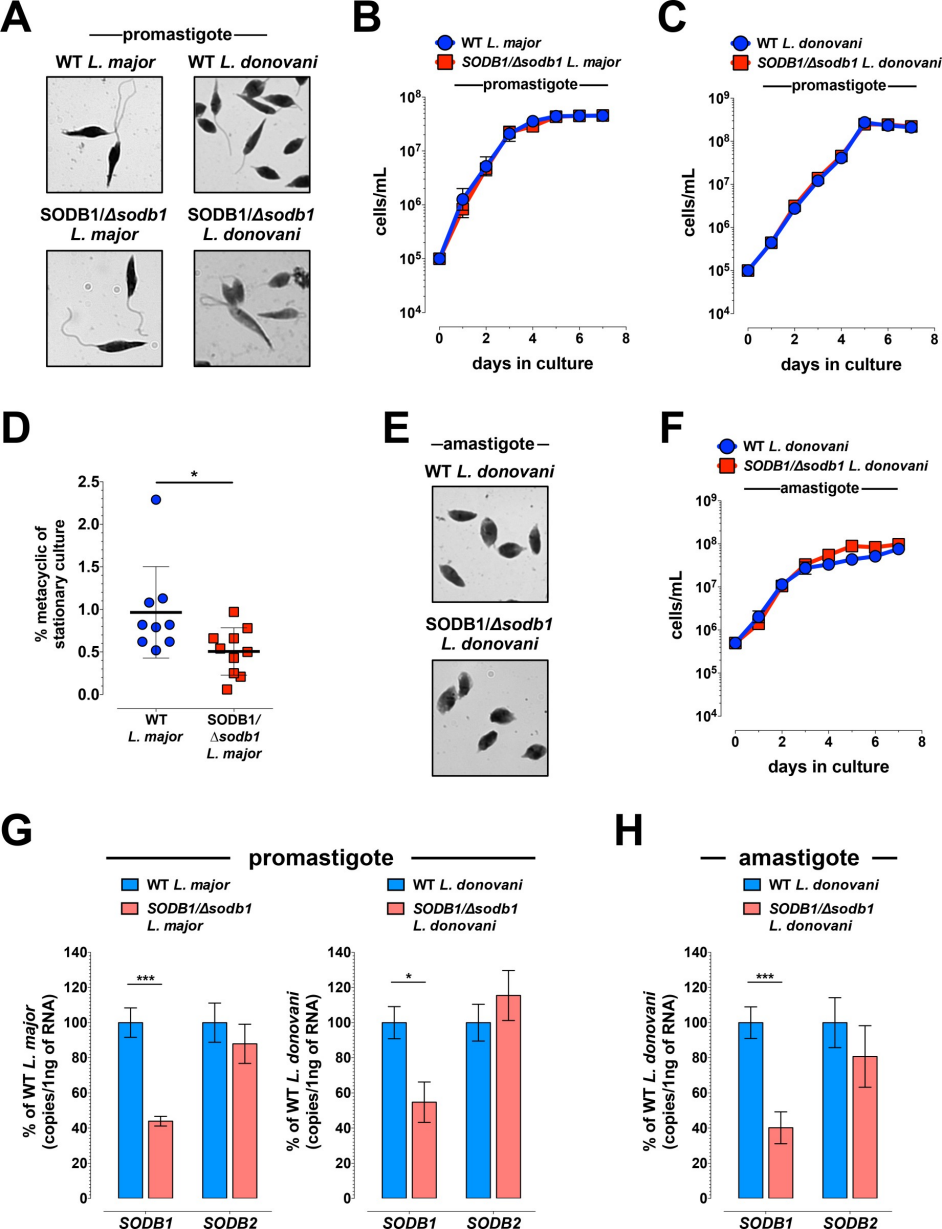
Deletion of a single *SODB1* allele does not affect growth of axenic *Leishmania* promastigotes or amastigotes in culture. (**A**) WT and *SODB1/Δsodb1 L*. *major* and *L*. *donovani* promastigotes were fixed and stained with Wright-Giemsa. Images show comparable promastigote morphology (magnification, 100x). (**B and C**) Triplicate cultures of WT and *SODB1/Δsodb1 L*. *major* (**B**) and *L*. *donovani* (**C**) promastigotes were seeded at 10^5^ cells per mL and counted daily by hemocytometer. Data are pooled from two independent experiments (n = 6/group). (**D**) Metacyclic WT and *SODB1/Δsodb1 L*. *major* promastigotes were purified by PNA agglutination and enumerated by hemocytometer (n = 9–10 per group). (**E**) WT and *SODB1/Δsodb1 L*. *donovani* amastigotes were fixed and stained with Wright-Giemsa. Images show comparable amastigote morphology (magnification, 100x). (**F**) Triplicate cultures of WT and *SODB1/Δsodb1 L*. *donovani* amastigotes were seeded at 5 x 10^5^ cells per mL and counted daily by hemocytometer. Data are pooled from three independent experiments (n = 9/group). (**G-H**) *SODB1* and *SODB2* expression levels were quantified by qRT-PCR in log-phase WT and *SODB1/Δsodb1 L*. *major* and *L*. *donovani* promastigotes (**G**) or WT and *SODB1/Δsodb1 L*. *donovani* amastigotes (**H**). Data are pooled from two independent experiments (n = 6/group). *P < 0.05, ***P < 0.001 by unpaired students *t*-test (**D, G and H**).

We next compared the properties of single allele *SODB1* knockout parasites (*SODB1/Δsodb1*) with WT parasites in culture. *SODB1/Δsodb1 L*. *major* and *L*. *donovani* promastigotes showed no gross morphological abnormalities compared with WT promastigotes (**Fig 1A**), and both *SODB1/Δsodb1 L*. *major* and *L*. *donovani* promastigotes grew at similar rates as WT in culture (**Fig 1B and 1C**). In addition, *SODB1/Δsodb1* and WT *L*. *major* parasites were capable of differentiating into infective metacyclic promastigotes. Nonetheless, *SODB1/Δsodb1* parasites showed a decrease (1.9-fold; *P* < 0.05) in the overall percentage of metacyclic promastigotes in stationary phase cultures (**Fig 1D**). These findings are consistent with previous analysis of *SODB1/Δsodb1 L*. *chagasi* promastigotes in which growth in culture did not deviate from WT promastigotes [12]. Next, *SODB1/Δsodb1 L*. *donovani* differentiation to amastigotes in response to low pH and increased temperature was evaluated [16]. Differentiation of promastigote forms to amastigotes and general morphology did not differ between WT and *SODB1/Δsodb1 L*. *donovani* (**Fig 1E**), and growth of parasites under axenic amastigote growth conditions was similar for WT and *SODB1/Δsodb1 L*. *donovani* (**Fig 1F**). To investigate if a single allele deficiency of *SODB1* enhanced *Leishmania* susceptibility to different oxidative stresses, we exposed parasites to increasing concentrations of H_2_O_2_ and menadione, which induces superoxide generation in mitochondria. Using a fixable dead cell stain-based viability assay and quantification by flow cytometry (**S2A and S2B Fig**), we found that WT and *SODB1/Δsodb1 L*. *major* or *L*. *donovani* promastigotes displayed similar sensitivities to both treatments (**S2C and S2D Fig**), suggesting that SODB1 is not essential for protection of late-log phase promastigotes against these oxidative stresses. Finally, to confirm that disruption of a single allele of *SODB1* reduced *SODB1* expression levels, we quantified *SODB1* RNA in log-phase promastigotes and in amastigotes. As shown in **Fig 1G and 1H**, we detected a 50–60% decrease of *SODB1* RNA in *SODB1/Δsodb1 L*. *major* and *L*. *donovani* promastigotes and *SODB1/Δsodb1 L*. *donovani* amastigotes compared with WT parasites. Importantly, these reductions were specific to RNA levels of *SODB1*, as the levels of *SODB2* RNA in *SODB1/Δsodb1* and WT parasites were similar (**Fig 1G and 1H**). Collectively, although the generation of *SODB1* null *L*. *major* or *L*. *donovani* parasites was not achieved by these methods, suggesting that SODB1 is vital for parasite survival, a single allele deletion does not alter general parasite morphology, promastigote growth *in vitro*, or, in the case of *L*. *donovani*, differentiation from promastigote to amastigote forms.

### Deletion of a single allele of *SODB1* impairs infection of macrophages

**Fig 2:**
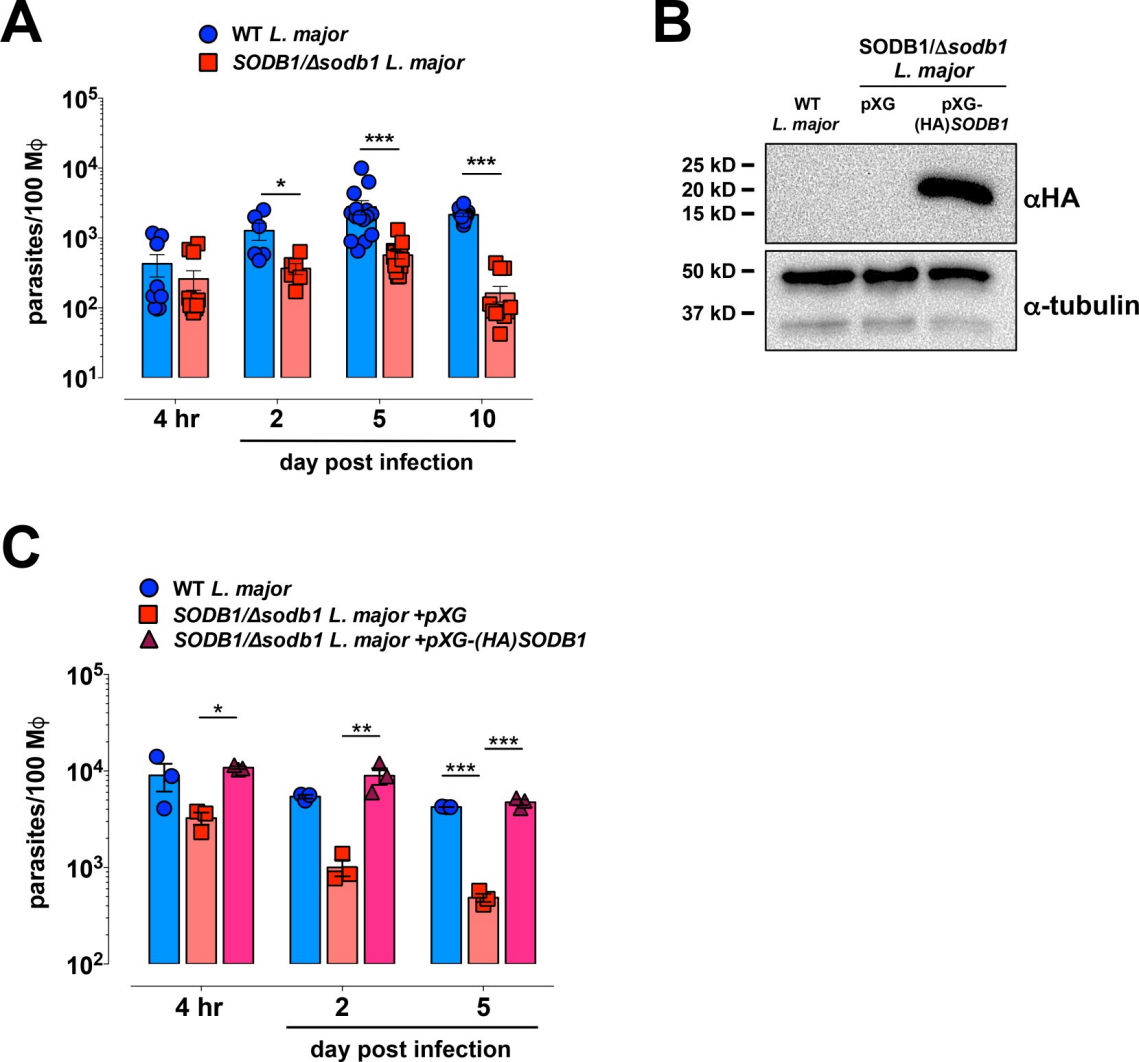
Deletion of a single allele of *SODB1* impairs infection of macrophages and is rescued through episomal *SODB1* complementation. (**A**) WT C57BL/6 BMDMs were infected at an MOI of 10 parasites/cell with WT or *SODB1/Δsodb1* stationary phase *L*. *major* promastigotes. At the times indicated, gDNA was isolated and total macrophage and *Leishmania* genomes were quantified by qPCR. Graphs represent the total *L*. *major* parasites per 100 BMDMs. Data are pooled from three independent experiments. (**B**) Anti-HA Western blot analysis of whole cell lysates from WT *L*. *major*, and *SODB1/Δsodb1 L*. *major* parasites complemented with pXG or pXG-(HA)-*SODB1*. Equal protein loading was determined by anti*-*α-tubulin Western blot analysis. (**C**) WT C57BL/6 BMDMs were infected at an MOI of 1 parasite/cell with purified metacyclic WT, *SODB1/Δsodb1* + pXG, or *SODB1/Δsodb1* + pXG-(HA)-*SODB1 L*. *major* parasites, and total parasites per 100 BMDMs were quantified by qPCR. Data are representative of two independent experiments. *P < 0.05, **P < 0.01, ***P < 0.001 by unpaired students *t*-test (**A**) or one-way ANOVA with Tukey’s multiple comparison test (**C**).

To evaluate the role of *SODB1* for infection in macrophages, BMDMs were generated from WT C57BL/6J mice. Macrophage cultures were inoculated with stationary phase WT or *SODB1/Δsodb1 L*. *major* promastigotes at an MOI of 10 parasites/cell. Total parasite genomes per 100 macrophages were quantified in infected cultures using qPCR assays specific for the *Leishmania ubiquitin hydrolase* gene and the murine *gapdh* gene. BMDMs infected with *SODB1/Δsodb1 L*. *major* promastigotes showed reduced parasite burdens on a per cell basis at 2, 5, and 10 day pi, but not at 4 h pi (**Fig 2A**). These data suggest that *SODB1* is dispensable for macrophage invasion but is required for intracellular replication and/or survival of *L*. *major* parasites in macrophages. To confirm that decreased *SODB1/Δsodb1 L*. *major* persistence in infected BMDMs is due to disrupted SODB1 expression, *SODB1/Δsodb1 L*. *major* parasites were complemented with a *Leishmania*-specific gene expression vector (pXG) bearing the complete *L*. *major SODB1* coding sequence. To enable detection of exogenous SODB1, an influenza virus HA sequence tag was added to the 5′-end of the *SODB1* gene in the pXG vector [12]. *SODB1/Δsodb1 L*. *major* promastigotes were transfected with either pXG or pXG-(HA)-*SODB1* and clones harboring the expression vector were isolated following drug selection. To confirm overexpression of HA-SODB1, whole cell lysates were evaluated by HA-specific western blot analysis. As shown in **Fig 2B**, *SODB1/Δsodb1 L*. *major* promastigotes transfected with pXG-(HA)-*SODB1*, but not empty pXG, express HA-tagged SODB1. BMDMs were infected with purified metacyclic promastigotes from WT, *SODB1/Δsodb1*+pXG and *SODB1/Δsodb1*+pXG-(HA)-*SODB1 L*. *major* promastigotes at an MOI of 1 parasite/cell. Quantification of total parasites per 100 macrophages revealed similar numbers of parasites at 2 and 5 day pi in macrophages infected with *SODB1/Δsodb1* parasites expressing HA-tagged SODB1 and WT parasites, whereas the number of parasites in macrophages infected with *SODB1/Δsodb1 L*. *major* plus the empty pXG vector were reduced (**Fig 2C**). These findings suggest that impaired persistence of *SODB1/Δsodb1* parasites in BMDMs is due to impaired SODB1 expression and can be rescued through episomal complementation with *SODB1*.

### Deletion of a single *SODB1* allele reduces the severity of lesions and tissue inflammation in mice

**Fig 3:**
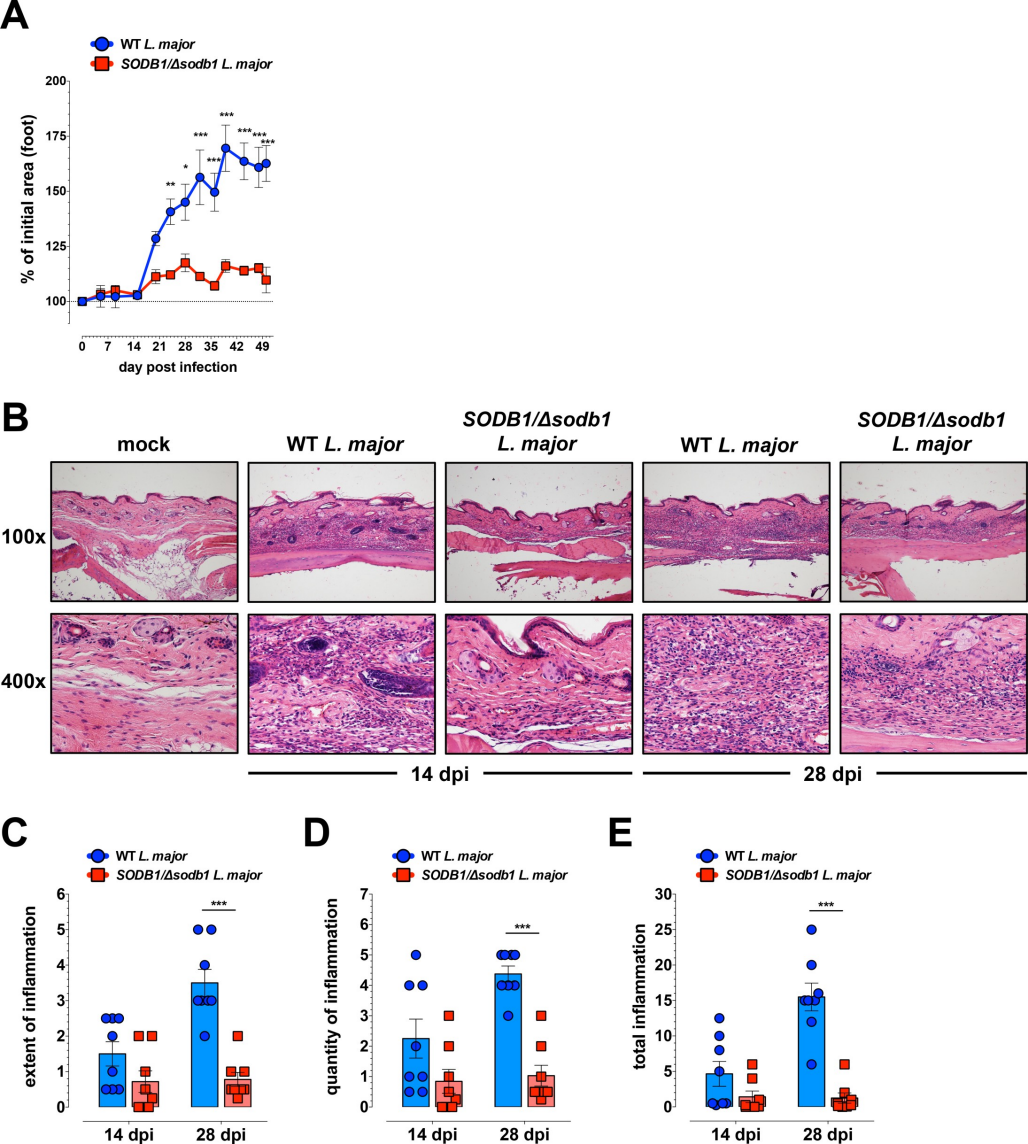
Deletion of a single *SODB1* allele impairs *L*. *major*-induced lesion development and inflammation in mice. Eight week-old WT C57BL/6 mice were inoculated on the dorsal surface subcutaneously in the right hind foot with 10^4^ WT or *SODB1/Δsodb1 L*. *major* purified metacyclic promastigotes. (**A**) Lesion development in the inoculated foot was measured over time by digital calipers and is presented as the % of initial foot area. Each data point represents the mean ± standard error of the mean (SEM) of 5 mice per group. Data are representative of 4 independent experiments. (**B**) At 14 and 28 dpi, hematoxylin and eosin-stained sections were prepared from paraffin-embedded foot tissues of uninfected mice (mock), and mice infected with WT or *SODB1/Δsodb1 L*. *major* metacyclic promastigotes. Images shown are of the region of highest inflammation within the tissue section. Images are representative of two independent experiments with 3–5 mice per group (n = 8 mice/group total). (**C-E**) Tissue sections were scored as described in the methods for the overall size (extent) of the inflammatory lesion (**C**), the density of inflammatory cells observed in the tissue (**D**), and total inflammation as a function of both the extent and density of inflammation. Data represent the mean ± SEM (**E**). *P < 0.05, **P < 0.01, ***P < 0.001 by 2-way ANOVA with Bonferroni’s multiple comparison test (**A and C-E**).

Disruption of a single *SODB1* allele in *L*. *major* parasites reduced infection levels over time in macrophages *in vitro*. To determine if *SODB1/Δsodb1 L*. *major* parasites exhibit altered pathogenicity *in vivo*, we utilized a mouse model of *Leishmania* pathogenesis. WT C57BL/6 mice infected with *L*. *major* parasites subcutaneously in the foot show disease signs such as lesion development over time, and contain measurable parasite burdens in the foot and the draining popLN [27,28]. We inoculated WT C57BL/6J mice with 10^4^ purified metacyclic WT or *SODB1/Δsodb1 L*. *major* promastigotes and monitored lesion development of the inoculated foot up to 50 dpi (**Fig 3A**). Mice infected with WT *L*. *major* exhibited disease progression as expected, with measurable lesion development first detectable between 14 and 21 dpi, and maximal lesion size attained by 40–42 dpi (**Fig 3A**). In contrast, mice infected with *SODB1/Δsodb1 L*. *major* metacyclic promastigotes showed significantly reduced lesion development as early as 24 dpi and failed to develop a temporal rise in lesion size up to 50 dpi (**Fig 3A**). To confirm these findings, we assessed the severity of inflammation within the foot tissue of mice infected with WT or *SODB1/Δsodb1 L*. *major* parasites. Examination of H&E stained foot tissue sections from WT and *SODB1/Δsodb1 L*. *major* infected mice at 14 and 28 dpi revealed an abundant inflammatory infiltrate composed of neutrophils, macrophages, and lesser numbers of lymphocytes throughout the full thickness of the dermis and hypodermis compared with mock-infected control mice (**Fig 3B and S3 Fig**). At 28 dpi, inflammation extended through the underlying fascia and muscle layers to and around the underlying periosteum that surrounds the metatarsals in all mice infected with WT *L*. *major*, but only a fraction of mice infected with *SODB1/Δsodb1 L*. *major* parasites (**Fig 3B**). Although mice infected with *SODB1/Δsodb1 L*. *major* showed similar infiltrate composition, both the size of the inflammatory lesion (extent of inflammation) and the density of inflammatory cells (quantity of inflammation) was reduced at 28 dpi (**Fig 3C and 3D)**. Quantification of total inflammation (the product of “extent” and “quantity” of inflammation) revealed that *SODB1/Δsodb1 L*. *major* infected mice had reduced inflammation at 28 dpi when compared with mice infected with WT *L*. *major* (**Fig 3E**). Lastly, the degree of inflammation in foot tissue of mice infected with WT *L*. *major* as measured by all criteria shows an increase between 14 and 28 dpi, whereas the degree of inflammation in mice infected with *SODB1/Δsodb1 L*. *major* remained similar between these time intervals (**Fig 3C–3E**). Collectively, these findings suggest that a normal level of SODB1 is essential for *L*. *major* virulence in WT C57BL/6 mice.

### Deletion of a single *SODB1* allele impairs L. major dissemination and persistence in WT mice

**Fig 4:**
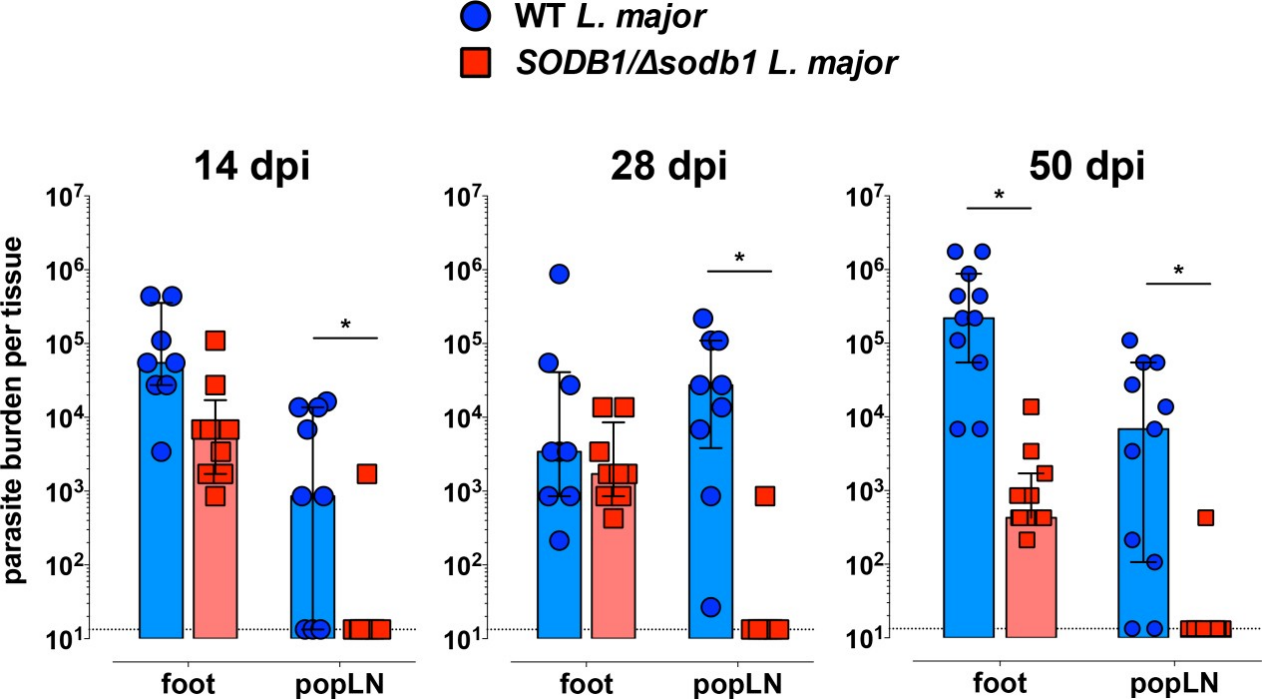
Deletion of a single *SODB1* allele reduces parasite dissemination and persistence in WT mice. Eight week-old WT C57BL/6 mice were inoculated subcutaneously in the right hind foot with 10^4^ WT or *SODB1/Δsodb1 L*. *major* purified metacyclic promastigotes. At 14, 28, and 50 dpi, parasite burdens in the right hind foot and draining popLN were quantified by limiting dilution assay. Data represent the mean ± standard error of the mean (SEM) and are pooled from 2–3 independent experiments per time point. *P < 0.05 by unpaired student’s *t*-test.

To determine if the decreased lesion development and severity observed in mice infected with *SODB1/Δsodb1 L*. *major* parasites was due to impaired infectivity or diminished parasite persistence, we performed a kinetic analysis of parasite burdens in foot tissue and the popLN, a primary site of parasite dissemination. At 14 and 28 dpi, parasite burdens in the foot were comparable in mice inoculated with WT and *SODB1/Δsodb1 L*. *major* metacyclic promastigotes, yet a lack of dissemination of *SODB1/Δsodb1* parasites to the draining popLN was evident at both time points post-infection (**Fig 4**). At 50 dpi, we detected a significant decrease in parasite burdens in the foot tissue of mice inoculated with *SODB1/Δsodb1* parasites compared with WT parasites, and a near absence of parasite infection in the popLN (**Fig 4**). Thus, the less severe inflammation and swelling observed in mice infected with *SODB1/Δsodb1 L*. *major* parasites is associated with a diminished capacity for *SODB1/Δsodb1 L*. *major* parasites to propagate or persist over time and to disseminate to tissues distal to the site of inoculation.

### *SODB1/Δsodb1 L. major* is attenuated in highly susceptible mouse strains

**Fig 5:**
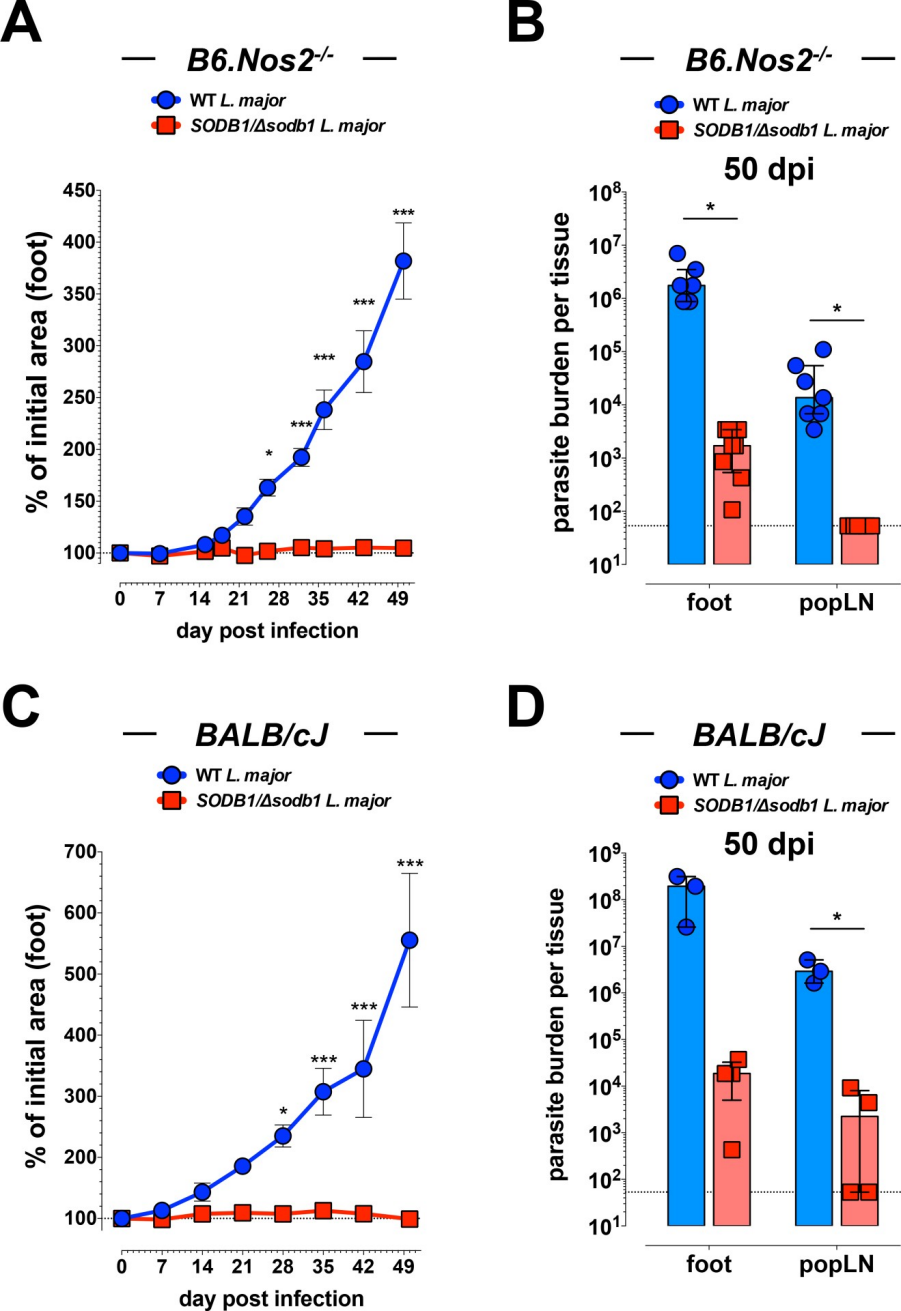
***SODB1/Δsodb1 L*. *major* parasites are attenuated in *Leishmania*-susceptible mouse strains** (**A and B**) Eight week-old *Nos2*^-/-^ C57BL/6 (*B6*.*Nos2*^*-/-*^) mice were inoculated in the dorsal subcutaneous tissue of the right hind foot with 10^4^ WT or *SODB1/Δsodb1 L*. *major* purified metacyclic promastigotes. (**A**) Lesion development in the inoculated foot was measured over time by digital calipers and is presented as the % of initial foot area. Each data point represents the mean ± standard error of the mean (SEM) of 4 mice per group. Data are representative of 2 independent experiments. (**B**) At 50 dpi, parasite burdens in the right hind foot and draining popLN were quantified by limiting dilution assay. Data represent the mean ± SEM and are pooled from 2 independent experiments. (**C and D**) Eight week-old WT Balb/cJ mice were inoculated subcutaneously in the right hind foot with 10^4^ WT or *SODB1/Δsodb1 L*. *major* purified metacyclic promastigotes. (**C**) Lesion development in the inoculated foot was measured over time by digital calipers and is presented as the % of initial foot area. Each data point represents the mean ± SEM of 5 mice per group. (**D**) At 50 dpi, parasite burdens in the right hind foot and draining popLN were quantified by limiting dilution assay. Data represent the mean ± SEM. *P < 0.05, ***P < 0.001 by 2-way ANOVA with Bonferroni’s multiple comparison test (**A and C**) or unpaired student’s *t*-test (**B and D**).

C57BL/6 mice deficient in inducible nitric oxide synthase (*B6*.*Nos2*^-/-^) and WT BALB/cJ mice are highly susceptible to *L*. *major* infection [29,30]. To determine if disruption of a single allele of *SODB1* in *L*. *major* is sufficient for attenuation in these highly susceptible mouse strains, we evaluated disease progression and parasite burdens in *Nos2*^-/-^ C57BL/6 mice and WT BALB/cJ mice infected with either 10^4^ purified WT or *SODB1/Δsodb1 L*. *major* metacyclic promastigotes. Similar to observations in WT C57BL/6 mice, *Nos2*^-/-^ C57BL/6 and WT BALB/cJ mice infected with WT *L*. *major* parasites developed severe lesions, with steady growth of lesion size out to 50 dpi. In contrast, *Nos2*^-/-^ C57BL/6 (*B6*.*Nos2*^-/-^) and WT BALB/cJ mice infected with *SODB1/Δsodb1 L*. *major* parasites showed little to no evidence of lesion development over time (**Fig 5A and 5C**). In addition to reduced lesion development, parasite burdens in the foot tissue and popLN of both mouse strains were reduced in mice infected with *SODB1/Δsodb1 L*. *major* compared with mice infected with WT *L*. *major* (**Fig 5B and 5D**). These findings provide further evidence that a normal level of SODB1 is required for *L*. *major* virulence in mice. Additionally, these data suggest that attenuation of *SODB1/Δsodb1 L*. *major* parasites is not due to heightened susceptibility to *Nos2*- or mouse strain-dependent effects.

## Discussion

An improved understanding of mechanisms by which *Leishmania* parasites replicate and persist in, and disperse from macrophages, particularly *in vivo*, may identify new therapeutic targets for the treatment of leishmaniasis. Pathogen-encoded SODs have known roles in detoxifying environmental superoxide as a countermeasure against host oxidative defenses. For example, in bacterial pathogens such as *Salmonella*, *Streptococcus* and *Mycobacterium*, periplasmic SODs confer protection against the oxidative burst generated by host neutrophils and macrophages, and in many cases is critical for promoting bacterial pathogenesis [31–35]. The genomes of *Leishmania* parasites are known to encode three superoxide dismutase genes: *SODA*, *SODB1*, and *SODB2* [12,36]. Notably, each of these SODs are targeted to membrane-bound organelles, with SODA targeted to mitochondria and SODB1 and SODB2 targeted to the glycosome. Recent studies implicated SODA in the regulation of *L*. *amazonensis* differentiation to infective forms, including both metacyclic promastigotes and amastigotes, by protecting against mitochondrial oxidative stress and activating ROS-dependent signaling. Moreover, disruption of a single *SODA* allele in *L*. *amazonensis* was sufficient to cause severe parasite attenuation in mice [21]. In contrast, no studies to date have evaluated the role of *SODB1* or *SODB2* in *Leishmania* pathogenesis *in vivo*.

Consistent with previous studies using other *Leishmania* species, we were unable to generate *SODB1* null *L*. *major* parasites using standard gene targeting approaches based on replacement of *SODB1* coding regions with a drug resistance gene by homologous recombination, suggesting that *SODB1* is essential for *L*. *major* viability [12]. Nevertheless, as heterozygous gene disruption of many *Leishmania* genes, including *SODA* [21], has revealed important insight into parasite biology, we evaluated the impact of disrupting a single *SODB1* allele on *L*. *major* and *L*. *donovani* differentiation to infective forms, infection of macrophages, and pathogenesis in mice.

During standard promastigote culture conditions, the morphology and growth rates of *SODB1/Δsodb1* and WT *L*. *major* and *L*. *donovani* promastigotes were comparable. In addition, *SODB1/Δsodb1 L*. *major* parasites differentiated into infective metacyclic promastigotes with nearly the same efficiency as WT parasites. Furthermore, the differentiation and axenic growth of *SODB1/Δsodb1 L*. *donovani* amastigotes also was comparable with WT parasites. Thus, in contrast to *SODA*, disruption of a single allele of *SODB1* had minimal impact on the growth and differentiation of *Leishmania* parasites in culture, suggesting that *Leishmania SODA and SODB1* genes have distinct functions [21]. This notion is further supported by the differential localization of SODA and SODB1 in the cell (mitochondria versus glycosome, respectively) [12,21,37,38]. However, as different *Leishmania* species were used in these studies, the impact of single allele deletions of *SODA* or *SODB1* on *L*. *major* parasite biology in culture remains to be confirmed.

Our data indicate that WT and *SODB1/Δsodb1 L*. *major* promastigotes have a similar capacity to establish infection in murine bone marrow macrophages. However, in contrast with WT *L*. *major* parasites, the number of *SODB1/Δsodb1 L*. *major* parasites in macrophages diminished over time, suggesting that a normal level of SODB1 is required for *L*. *major* persistence in macrophages. Complementation of *SODB1/Δsodb1 L*. *major* parasites with episomal expression of SODB1 restored parasite numbers in macrophages to levels equivalent with WT *L*. *major*, further suggesting that SODB1 promotes the survival of *L*. *major* parasites in macrophages.

Due to the impaired capacity of *SODB1/Δsodb1 L*. *major* parasites to persist in murine macrophages, we evaluated the effects of a single allele deletion of *SODB1* on virulence and persistence in mice. WT C57BL/6 mice infected with *SODB1/Δsodb1 L*. *major* purified metacyclic promastigotes developed mild cutaneous lesions compared with mice infected with WT *L*. *major* metacyclic promastigotes, which displayed normal lesion progression. Similar outcomes were observed in more susceptible *Nos2*^-/-^ C57BL/6 and WT BALB/c mice, suggesting that *SODB1/Δsodb1 L*. *major* parasites are not more susceptible to nitric oxide produced from inducible NOS (iNOS; Nos2) commonly expressed in macrophages and neutrophils. Although additional functional verification studies are ongoing, these findings suggest that SODB1 is essential for *L*. *major* virulence in mice. In WT C57BL/6 mice, the burdens of WT and *SODB1/Δsodb1 L*. *major* parasites in the inoculated foot were comparable at both 14 and 28 dpi. In contrast, by 50 dpi, parasite burdens in the foot tissue of WT and *Nos2*^-/-^ C57BL/6 mice, as well as WT BALB/c mice, infected with *SODB1/Δsodb1 L*. *major* were strongly reduced compared with WT *L*. *major* burdens. Similar to our observations in bone marrow macrophages, these data suggest that a normal level of SODB1 is dispensable for *L*. *major* to establish infection, but essential for long-term *L*. *major* persistence. We also found that *SODB1/Δsodb1 L*. *major* parasite dissemination to the draining popLN was virtually absent throughout the time course evaluated. In mice, activation of innate immune responses during *Leishmania* infection are critical for curtailing parasite spread to organs distal to the site of inoculation [39]. Thus, the diminished capacity of *SODB1/Δsodb1 L*. *major* parasites to disseminate to the draining popLN suggests that *SODB1/Δsodb1 L*. *major* parasites are more susceptible to host innate defenses.

The reduced capacity for *SODB1/Δsodb1 L*. *major* parasites to persist in macrophages may be due to critical roles for SODB1 in detoxification of superoxide produced in the glycosome, to enhanced susceptibility to host oxidative defenses, or both. The glycosome is a membrane bound organelle uniquely found in trypanosomatid parasites that compartmentalizes enzymes involved in glycolysis [14]. Thus, the localization of SODB1 to this internal organelle, the proper function of which is required for parasite survival, the attenuation of *SODB1/Δsodb1 L*. *major* parasites in *Nos2*^-/-^ mice, and the decreased survival of *SODB1/Δsodb1 L*. *chagasi* parasites exposed to paraquat [12], suggests important roles for SODB1 in detoxification of endogenous superoxide. However, it remains possible that SODB1 also influences *Leishmania* susceptibility to exogenous reactive oxygen species. Experiments are ongoing to evaluate both of these possibilities.

Our findings expand upon previous studies suggesting that *Leishmania* glycosomal SODB1 protects from exogenous reactive oxygen species and promotes parasite survival in macrophages *in vitro* [12,15]. Here, we show that a single allele deletion in the *L*. *major SODB1* gene leads to significant attenuation of disease severity, as well as parasite dissemination and persistence in mice. Therefore, therapeutic disruption of SODB1 function may provide an efficacious means for mitigating leishmaniasis.

## Supporting information

S1 Table Nucleotide sequences for primers used to generate targeting and overexpression constructs, and for ubiquitin hydrolase and gapdh qPCR. (DOCX)

S1 Fig Deletion of a single *SODB1* allele in *L. major* and *L. donovani*. (**A**) Schematic of the *SODB1* gene targeting strategy. The intergenic regions (IGR) upstream (5′ IGR) and downstream (3′ IGR) of the *SODB1* coding region included in the targeting construct are shown. The targeting strategy is designed to replace the *SODB1* coding region with the coding region of neomycin (*NEO*) or phleomycin (*PHLEO*) resistance genes. Arrows represent PCR primers used for screening, and relative sizes of PCR products from *L*. *major* (*Lm*) or *L*. *donovani* (*Ld*) WT, or targeted *SODB1* alleles is indicated. (**B and C**) PCR products from the endogenous *SODB1* WT locus or unique to precise integration of the targeting construct were amplified from gDNA of *L*. *major* (**B**) or *L*. *donovani* (**C**) WT (*wt*) or *SODB1/Δsodb1* (*mut*) parasites. A no template control (*neg*) is included. Pr1+Pr2 primer combinations (5′ amplification of *SODB1* targeted allele) generated two faint products from WT *L*. *major* (**B**), which are both either smaller or larger than the product generated from construct targeting of *SODB1*, and likely represents spurious amplification events that may occur in a PCR reaction that lacks primer-specific targets. PCR products were sequenced to verify the border regions between the endogenous genetic locus and the targeting construct bearing *NEO* or *PHLEO* drug cassettes.
(TIF)

S2 Fig Parasite susceptibility to oxidative stress *in vitro*. (**A**) Late log-phase WT *L*. *major* promastigotes were untreated (untx) or heated at 56°C for the indicated times. Following heat treatment, parasite viability was quantified by flow cytometry using an AquaVi-42-based viability assay described in the Methods. (**B**) Late log-phase WT *L*. *major* promastigotes were exposed to vehicle only or H_2_O_2_ (2 mM) for 4 h and parasite viability was quantified by an AquaVi-421-based viability assay. All plots in (**A**) and (**B**) are gated on parasites by FSC/SSC, and the numbers indicate % AquaVi-421^+^ among all parasites. (**C**) Late log-phase WT or *SODB1/Δsodb1 L*. *major* and *L*. *donovani* promastigotes were treated with vehicle only or graded doses of H_2_O_2_ for 4 h and parasite viability was quantified by an AquaVi-421-based viability assay. (**D**) Following 6 days of exposure to vehicle or graded doses of menadione, WT or *SODB1/Δsodb1 L*. *major* and *L*. *donovani* promastigotes were assessed for viability by an AquaVi-421-based viability assay. Parasite viability in (**C**) and (**D**) is presented as the % viable of vehicle-treated controls. Data are presented as mean ± SEM. **P < 0.01, ***P < 0.001 by two-way ANOVA with Bonferroni’s multiple comparison test.
(TIF)

S3 Fig Inflammatory infiltrate in infected foot tissue consists of macrophages, neutrophils, and lymphocytes. H&E stained foot tissue at 28 dpi from mice infected with WT *L*. *major* (top panel) or *SODB1*/*Δsodb1 L*. *major* (bottom panel) (magnification, 400x). Infiltrating neutrophils (white arrows), macrophages (black arrows), and lymphocytes (grey arrows) are highlighted.
(TIF)
