# Supplementary material for: SODB1 is essential for Leishmania major infection of macrophages and pathogenesis in mice
Source: PLoS Negl Trop Dis. 2018 Oct 29;12(10):e0006921. doi: 10.1371/journal.pntd.0006921 (PMC6224164; doi:10.1371/journal.pntd.0006921)
Supplement: S1 Table — (DOCX) [file pntd.0006921.s001.docx]

| **Table S1.** Nucleotide sequences for primers used to generate targeting and overexpression contructs, *ubiquitin hydrolase* and *gapdh* qPCR, and *Leishmania* *SODB1* and *SODB2* qRT-PCR. | | | |
| --- | --- | --- | --- |
| ***L. major* *SODB1* targeting construct primers**^§^**^∗^** | | | |
|  |  |  |  |
| Lmaj sodb1 5'arm XhoI fwd | 5' | ACTG*CTCGAG***GCCGGGCTGATCTATATCCG** | 3' |
| Lmaj sodb1 5'arm BamHI rev | 5' | ACTG*GGATCC***GACGCACGAACAGAAAGTAA** | 3' |
| Lmaj sodb1 3'arm BamHI fwd | 5' | ACTG*GGATCC***GGTTGTCTGCGATACACAGTTTGG** | 3' |
| Lmaj sodb1 3'arm NotI fwd | 5' | ACTG*GCGGCCGC***CACACCTCTGCAACGACAGC** | 3' |
|  |  |  |  |
| ***L. major* Δ*sodb1* screening primers** | | | |
|  |  |  |  |
| Lmaj Δsodb1 seq 5' arm fwd | 5' | **GTACAACGTACACAACTCAC** | 3' |
| Lmaj Δsodb1 seq 3' arm rev | 5' | **CTTCTTGCTCCCACGAGCGC** | 3' |
| Pr1 | 5' | **GTCGAGCGAAAGAGATGTCG** | 3' |
| Pr2 | 5' | **CACGGCGGCATCAGAGCAGC** | 3' |
| Pr3 | 5' | **GCGCATCGCCTTCTATCGCC** | 3' |
| Pr4 | 5' | **GGCAAAGACACAAGCGAGCG** | 3' |
| Pr5 | 5' | **GACTACAAGAACGACCGCGC** | 3' |
| Pr6 | 5' | **CACTGTCGCAGAGACAGACG** | 3' |
|  |  |  |  |
| ***L. donovani* *SODB1* targeting construct primers (Gibson assembly)** | | | |
|  |  |  |  |
| Ldon sodb1 5'arm fwd | 5' | cgaattcctgcagcccgggg**GCTGGTGCGCTGTGGGAT** | 3' |
| Ldon sodb1 5'arm rev | 5' | ccgatcccat**GACGCACGAGCAGAAAGC** | 3' |
| Ldon sodb1 3'arm fwd | 5' | gttcttctga**GCTCTTCTGCGATGCGCA** | 3' |
| Ldon sodb1 3'arm rev | 5' | cggccgctctagaactagtg**GTCCATCATGTCCATCACGC** | 3' |
| Ldon neo fwd | 5' | **CTCGTGCGTC**ATGGGATCGGCCATTGAAC | 3' |
| Ldon neo rev | 5' | **GCAGAAGAGC**TCAGAAGAACTCGTCAAGAAG | 3' |
|  |  |  |  |
| ***L. donovani* Δ*sodb1* screening primers** | | | |
|  |  |  |  |
| Ldon 5'ArmB1 seq fwd | 5' | **CAGCTAAGGAATCGGACGACG** | 3' |
| Ldon 3'ArmB1 seq rev | 5' | **TGAACTCCAAACTGCGCATC** | 3' |
| Ldon Δsodb1 seq 5' arm (Pr1) | 5' | **GTCGGGTGATGCTTGACTGTCG** | 3' |
| Ldon Δsodb1 seq 3' arm (Pr5) | 5' | **GATCTTGACGTGCGATATATGG** | 3' |
| Ldon Δsodb1 seq 3' arm (Pr6) | 5' | **CGCAAGCATGAGCACACGGC** | 3' |
| Ldon Δsodb1 seq 3' arm (Pr4) | 5' | **GTCAATACCATCTCGTCATG** | 3' |
|  |  |  |  |
| ***L. major* *ubiquitin hydrolase* gDNA qPCR primers and probe** | | | |
|  |  |  |  |
| UbHyd qPCR fwd | 5' | **GCGACGGAGTCTATGTATGTATG** | 3' |
| UbHyd qPCR rev | 5' | **GAGCTTCTTGACGGTTGTGTA** | 3' |
| UbHyd qPCR probe | 5' | (5'6-FAM) **GAGCACCTGGAGAAGCACATGAGG** (3'BlackHoleQuencher 2) | 3' |
|  |  |  |  |
| **Murine *gapdh* gDNA qPCR primers and probe** | | | |
|  |  |  |  |
| GAPDH fwd | 5' | CAGTGACTTGGGACAAGGATAG | 3' |
| GAPDH rev | 5' | TCCTCAGTGTAGCCCAAGA | 3' |
| GAPDH probe | 5' | (5'6-FAM) TCAAGAAGGTGGTGAAGCAGGCAT (3'BlackHoleQuencher 2) | 3' |
|  |  |  |  |
| ***L. major* *SODB1* cDNA HA tag primers** | | | |
|  |  |  |  |
| Lmaj *(HA)SODB1* SmaI fwd | 5' | ACTG*CCCGGG*ATGTACCCATACGATGTTCCAGATTACGCT**CCGTTCGCTGTTCAG** | 3' |
| Lmaj *SODB1* BamHI rev | 5' | ACTG*GGATCC***CTAAAGCTGGCTAGAGGCG** | 3' |
|  |  |  |  |
| ***L. major* & *L. donovani SODB1/SODB2* qRT-PCR primers and probe** | | |  |
|  |  |  |  |
| Lmaj/Ldon sodb1/2 qRT fwd | 5' | **GGTGGACATCATCAAGTCTGAGAAG** | 3' |
| Lmaj sodb1 qRT rev | 5' | **ATTGCCCGATGGCTCGC** | 3' |
| Lmaj sodb2 qRT rev | 5' | **TCTCCGTGCGGTTTTGAC** | 3' |
| Ldon sodb1 qRT rev | 5' | **GCACTCGCCAAAGGGCCCGA** | 3' |
| Ldon sodb2 qRT rev | 5' | **ATCTCTCCGTGCGGCTTCGAC** | 3' |
| Lmaj/Ldon sodb1/2 probe | 5' | (5'6-FAM) **TACAACCACGACTTCTTCTGGCGC** (3'BlackHoleQuencher 2) | 3' |
|  |  |  |  |
| § *Italicized* and underlined nucleotides = restriction sites | | |  |
| ∗**Bold** nucleotides = *L. major* and *L. donovani* genomic sequences | | |  |
| Red nucleotides = influenza HA tag sequence | | |  |
