# Supplementary figures and images for: SODB1 is essential for Leishmania major infection of macrophages and pathogenesis in mice
Source: PLoS Negl Trop Dis. 2018 Oct 29;12(10):e0006921. doi: 10.1371/journal.pntd.0006921 (PMC6224164; doi:10.1371/journal.pntd.0006921)

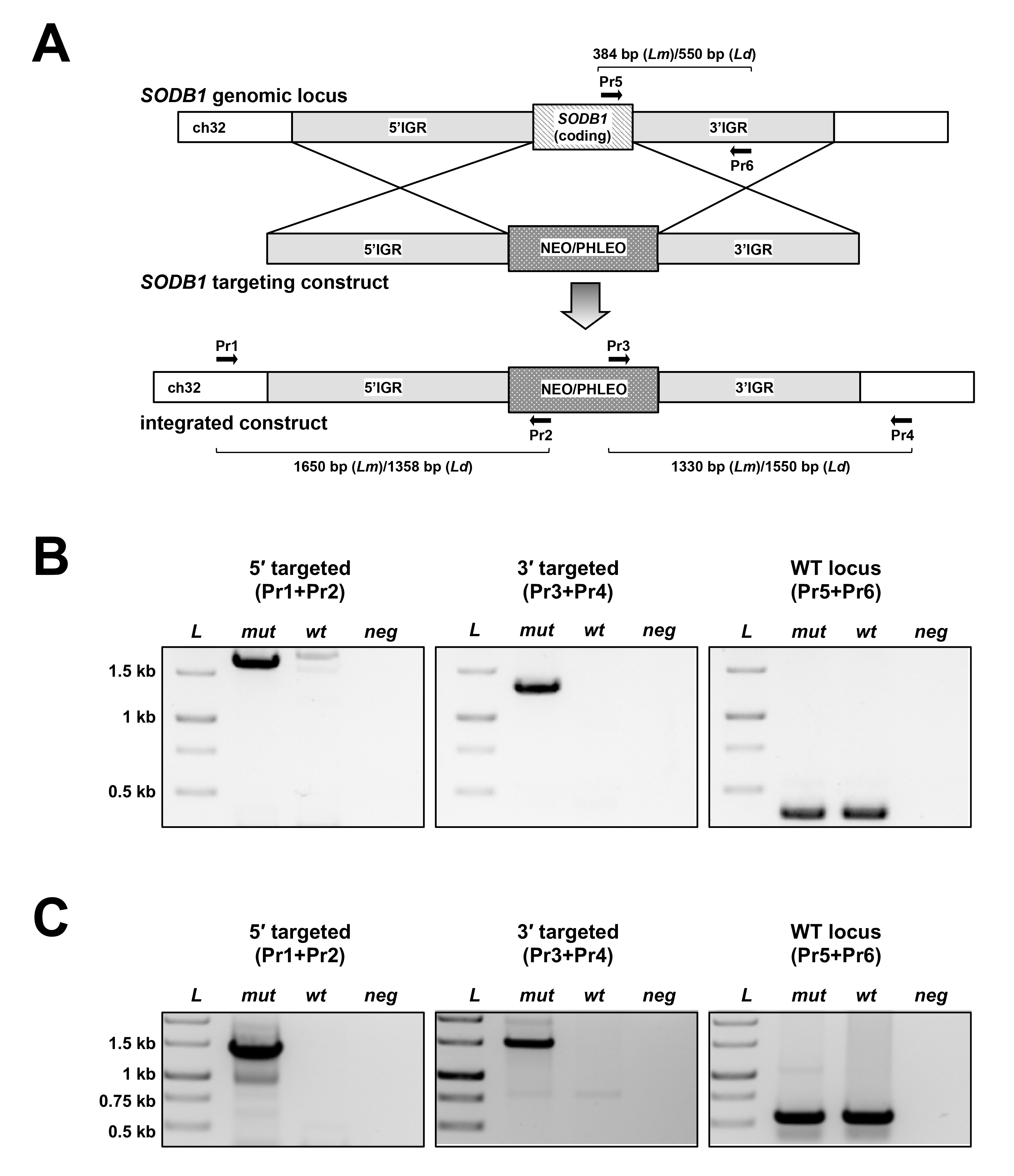

Supplement: S1 Fig — (A) Schematic of the SODB1 gene targeting strategy. The intergenic regions (IGR) upstream (5′ IGR) and downstream (3′ IGR) of the SODB1 coding region included in the targeting construct are shown. The targeting strategy is designed to replace the SODB1 coding region with the coding region of neomycin (NEO) or phleomycin (PHLEO) resistance genes. Arrows represent PCR primers used for screening, and relative sizes of PCR products from L. major (Lm) or L. donovani (Ld) WT, or targeted SODB1 alleles is indicated. (B and C) PCR products from the endogenous SODB1 WT locus or unique to precise integration of the targeting construct were amplified from gDNA of L. major (B) or L. donovani (C) WT (wt) or SODB1/Δsodb1 (mut) parasites. A no template control (neg) is included. Pr1+Pr2 primer combinations (5′ amplification of SODB1 targeted allele) generated two faint products from WT L. major (B), which are both either smaller or larger than the product generated from construct targeting of SODB1, and likely represents spurious amplification events that may occur in a PCR reaction that lacks primer-specific targets. PCR products were sequenced to verify the border regions between the endogenous genetic locus and the targeting construct bearing NEO or PHLEO drug cassettes. (TIF) [file pntd.0006921.s002.tif]

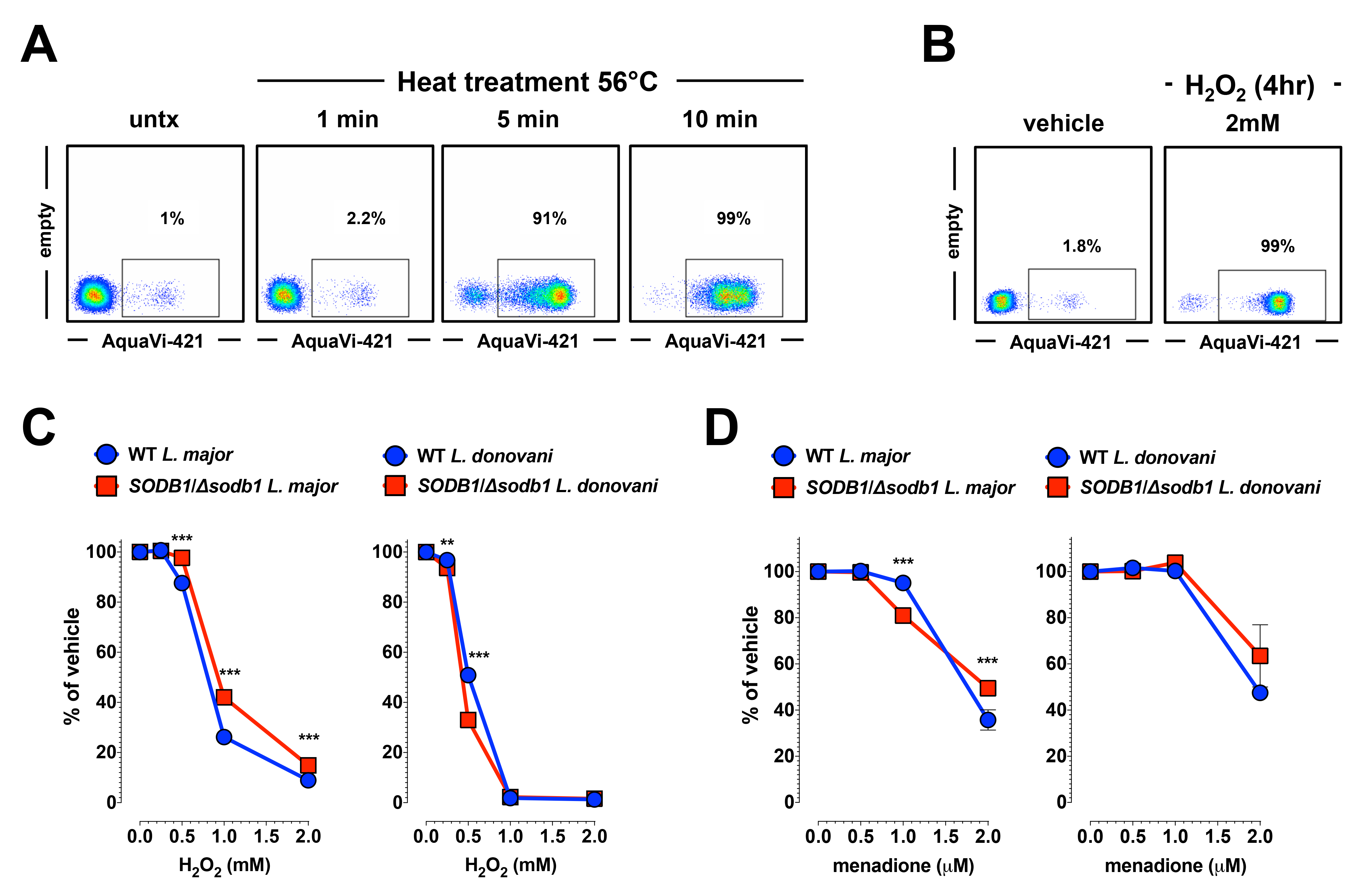

Supplement: S2 Fig — (A) Late log-phase WT L. major promastigotes were untreated (untx) or heated at 56°C for the indicated times. Following heat treatment, parasite viability was quantified by flow cytometry using an AquaVi-42-based viability assay described in the Methods. (B) Late log-phase WT L. major promastigotes were exposed to vehicle only or H2O2 (2 mM) for 4 h and parasite viability was quantified by an AquaVi-421-based viability assay. All plots in (A) and (B) are gated on parasites by FSC/SSC, and the numbers indicate % AquaVi-421+ among all parasites. (C) Late log-phase WT or SODB1/Δsodb1 L. major and L. donovani promastigotes were treated with vehicle only or graded doses of H2O2 for 4 h and parasite viability was quantified by an AquaVi-421-based viability assay. (D) Following 6 days of exposure to vehicle or graded doses of menadione, WT or SODB1/Δsodb1 L. major and L. donovani promastigotes were assessed for viability by an AquaVi-421-based viability assay. Parasite viability in (C) and (D) is presented as the % viable of vehicle-treated controls. Data are presented as mean ± SEM. **P < 0.01, ***P < 0.001 by two-way ANOVA with Bonferroni’s multiple comparison test. (TIF) [file pntd.0006921.s003.tif]

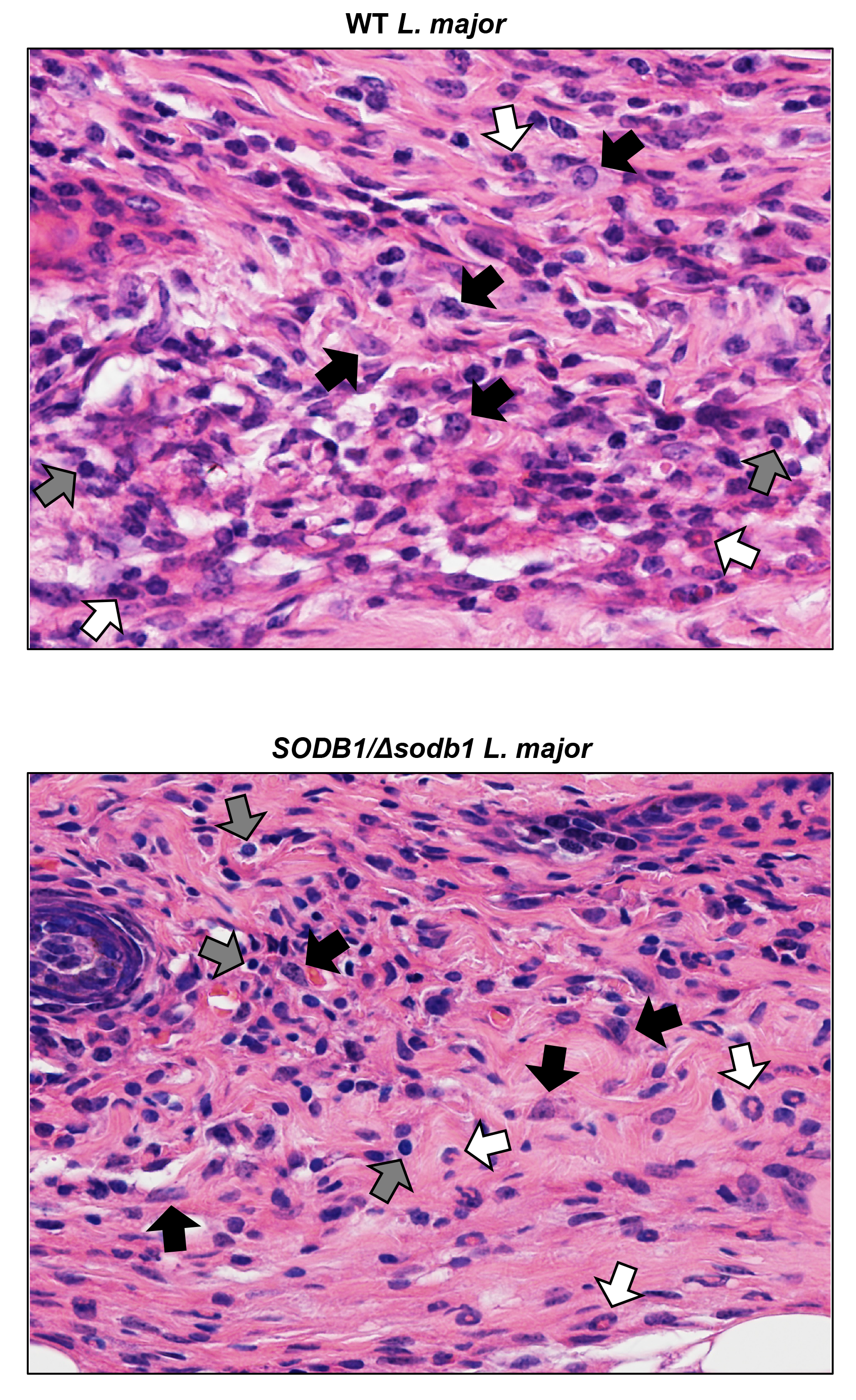

Supplement: S3 Fig — H&E stained foot tissue at 28 dpi from mice infected with WT L. major (top panel) or SODB1/Δsodb1 L. major (bottom panel) (magnification, 400x). Infiltrating neutrophils (white arrows), macrophages (black arrows), and lymphocytes (grey arrows) are highlighted. (TIF) [file pntd.0006921.s004.tif]
